# Chiropractic Manipulation Increases Maximal Bite Force in Healthy Individuals

**DOI:** 10.3390/brainsci8050076

**Published:** 2018-04-27

**Authors:** Heidi Haavik, Mustafa Görkem Özyurt, Imran Khan Niazi, Kelly Holt, Rasmus Wiberg Nedergaard, Gizem Yilmaz, Kemal Sitki Türker

**Affiliations:** 1 Centre for Chiropractic Research, New Zealand College of Chiropractic, 1060 Auckland, New Zealand; heidi.haavik@nzchiro.co.nz (H.H.); kelly.holt@nzchiro.co.nz (K.H.); Rasmus.nedergaard@nzchiro.co.nz (R.W.N.); 2 School of Medicine, Koç University, 34450 Istanbul, Turkey; gozyurt14@ku.edu.tr (M.G.Ö.); giyilmaz@ku.edu.tr (G.Y.); 3 Centre for Chiropractic Research, New Zealand College of Chiropractic, 1060 Auckland, New Zealand; imran.niazi@nzchiro.co.nz; 4 Health & Rehabilitation Research Institute, Auckland University of Technology, 1142 Auckland, New Zealand; 5 SMI, Department of Health Science and Technology, Aalborg University, DK-9220 Aalborg, Denmark; 6 Dr. Sid E. Williams Center for Chiropractic Research, Life University, Marietta, GA 30060, USA

**Keywords:** total maximal bite force, chiropractic care, spinal manipulation

## Abstract

Recent research has shown that chiropractic spinal manipulation can alter central sensorimotor integration and motor cortical drive to human voluntary muscles of the upper and lower limb. The aim of this paper was to explore whether spinal manipulation could also influence maximal bite force. Twenty-eight people were divided into two groups of 14, one that received chiropractic care and one that received sham chiropractic care. All subjects were naive to chiropractic. Maximum bite force was assessed pre- and post-intervention and at 1-week follow up. Bite force in the chiropractic group increased compared to the control group (*p* = 0.02) post-intervention and this between-group difference was also present at the 1-week follow-up (*p* < 0.01). Bite force in the chiropractic group increased significantly by 11.0% (±18.6%) post-intervention (*p* = 0.04) and remained increased by 13.0% (±12.9%, *p* = 0.04) at the 1 week follow up. Bite force did not change significantly in the control group immediately after the intervention (−2.3 ± 9.0%, *p* = 0.20), and decreased by 6.3% (±3.4%, *p* = 0.01) at the 1-week follow-up. These results indicate that chiropractic spinal manipulation can increase maximal bite force.

## 1. Introduction

Multiple studies over the past two decades have suggested that spinal manipulation can alter cortical sensorimotor integration, motor control and strength of voluntary human muscles [1,2,3,4,5,6]. In particular, increases in maximum voluntary force have been shown in lower limb muscles following spinal manipulation [4,6]. Only a small significant change in the H-reflex has been observed in healthy subjects [4], while large changes in cortical drive (as measured with the V-wave) have accompanied the strength increases seen in both a healthy population and elite sports performers [4,6]. This supports the hypothesis that chiropractic spinal manipulation of dysfunctional spinal segments leads to central neural plastic changes that occur to a large extent at the supraspinal level [1]. If there is a real central effect, then it should also be possible to measure changes in cranial nerve-innervated structures such as the jaw. From chiropractic studies, there are mainly case studies that indicate chiropractic care may help some people with trigeminal neuralgia [7], although one group has shown that spinal manipulation can increase jaw opening and increase pressure pain thresholds over a trigeminal nerve distribution area (sphenoid bone) in women with mechanical neck pain [8]. 

A review of the literature in 2006 explored any potential association between the cervical spine, the stomatognathic system, and craniofacial pain [9]. The conclusion of this review was that the current literature suggests an association between the cervical spine, the stomatognathic system, and craniofacial pain, but the authors noted that it was not conclusive as most of the literature they had reviewed was derived from poor quality studies [9]. Further research is therefore needed to understand what influence, if any, the cervical spine has on jaw function. 

The maximal biting force of various animals, including humans, has been investigated using different techniques. Researchers usually use two or more teeth to measure bite force in humans [10,11,12]. Ideally, however, due to the increased number of periodontal receptor involvement, it is best to measure total bite force where all the teeth can contribute [13,14]. 

Although the head, neck, and jaw are linked biomechanically and neurologically [15,16] there is still little evidence regarding the impact of changing somatosensory input from the neck on jaw muscle function. Previous research has shown that by altering the sensory input this can alter jaw muscles activity [17]. In several animal studies, reduced facilitation of the masseter muscle was shown when the sensory feedback from the periodontal receptors was removed, indicating the importance of the peripheral feedback systems in controlling masticatory muscles activity [13,14]. We also know that jaw function is often coupled with neck muscle activation. For example, there is co-contraction of the neck muscles observed during jaw clenching [18], and contraction of the masseter muscle is also increased when the trapezius and sternomastoid muscles are activated, suggesting a common motor control mechanism for maintaining neck and head stability together [19,20,21,22,23]. 

It is, therefore, possible that cervical spine dysfunction or cervical spinal manipulation of dysfunctional segments may also impact jaw muscle motor control. We propose that spinal manipulation will alter the somatosensory feedback from the deep paraspinal musculature around the dysfunctional spinal segments that get manipulated and that this altered feedback may also impact jaw muscle function, as measured with maximum jaw muscle strength. The aim of this study was, therefore, to investigate whether chiropractic spinal manipulation could impact maximal bite force.

## 2. Materials and Methods

After approval from the Committee on Ethics at Koç University (protocol number: 2013.066.IRB2.33/11.02.2013, re-approved in 2018), 28 people between the ages of 18 and 65 participated in a two-arm randomized parallel group study design with one week follow up, after filling the informed consent form. The setting for the study was a laboratory at Koç University, Istanbul, Turkey. All the experiments were performed in accordance with the rules of the Declaration of Helsinki. Both study arms had equal (*N* = 14) subjects for the first session (intervention or control, described below) but for the follow-up session, only 6 subjects were able to return for each group because of time limitations for completing data collection. 

**Figure 1:**
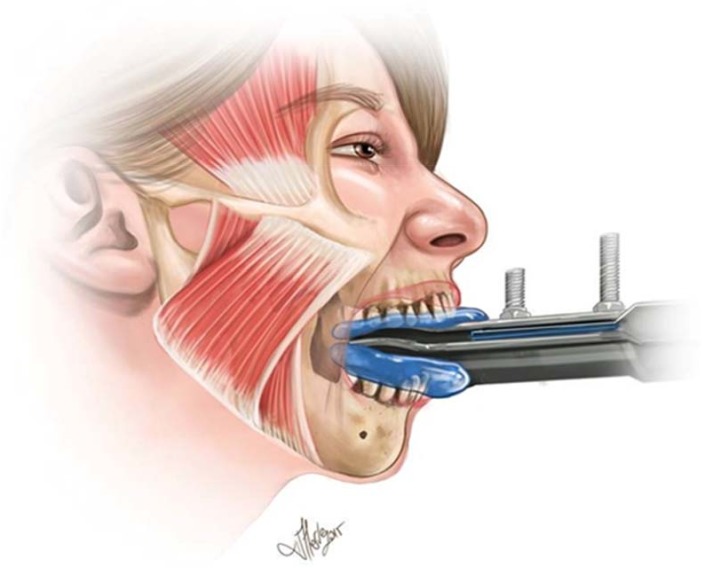
The illustration depicts the biting platform and the position of the subject. The mold illustrated as blue, allowed all teeth to be involved. A force transducer was placed close to the base of the tungsten plates which corresponds to the right most of the illustration.

To obtain maximal bite force, a strain gauge was placed under tungsten bite plates. Two bite plates were fixed about 1 cm apart. Express STD vinyl polysiloxane impression material was used as a mold with a 1:1 ratio of base and its catalyst. This mixture was glued on both sides of the tungsten plates which allowed all teeth to take part in the bite attempt. This mold also reduced the risk of tooth injury during maximal bite force attempts. The system is illustrated in Figure 1.

The strain gauges were placed in the inner part of the bite plates. Subject’s teeth were embedded in the mold so that all his/her teeth contributed to the total bite force. In each session, the subject was asked to bite as much as s/he could with verbal encouragement from one of the researchers. Each subject bit the mold three times for 3 s with an interval of 60 s between each bite attempt. Subjects were randomly allocated to either spinal manipulation or a sham intervention, however, the subjects were all told they were going to receive a chiropractic intervention as all subjects were naïve to chiropractic care due to the limited number of chiropractors practicing in Turkey. Bite force was assessed pre- and post the intervention and at 1-week follow up. After their last assessment session, the participants were informed whether they received the sham or chiropractic care. Elven out of 14 in the sham group believed they received the chiropractic care.

### 2.1. Intervention

#### 2.1.1. Spinal Manipulation 

The chiropractic intervention consisted of spinal manipulation of dysfunctional spinal and sacroiliac joints (also known within the chiropractic profession as subluxations). The entire spine and pelvis were assessed for segmental dysfunction by a chiropractor with over 10 years of clinical experience and multiple levels and regions were adjusted in each participant. Clinical indicators used to assess for dysfunction were; manually palpating for restricted inter-segmental motion, tenderness to palpation over relevant joints, any abnormal or blocked joint play, palpable asymmetric inter-vertebral muscle tension. These assessment methods are used by the chiropractic profession and other manual therapists as indicators of spinal dysfunction [24]. The spinal manipulations were all high-velocity low amplitude thrusts to the dysfunctional vertebral segments. The mechanical properties of this type of spinal manipulation have been investigated, and although the actual force applied to the spine depends on the clinician, the general shape of the force-time history is very consistent [25]. This spinal manipulation technique has been previously employed by our research group to investigate the neurophysiologic effects of spinal manipulation [1,4,5].

#### 2.1.2. Sham Intervention

The control intervention consisted of passive motion of the spine and moving the participant into a position that a chiropractor would normally deliver the spinal thrusts (i.e., a pre-manipulation position). This was performed by one of the researchers who was not trained as a chiropractor and care were taken not to load any particular spinal joints to end range as this is known to alter proprioceptive firing of the paraspinal tissues in anesthetized cats [26]. No manual thrust was performed during the control intervention. In this situation, with subjects who were naïve to chiropractic care, it was felt that the control intervention would act as a reasonable sham, while also controlling for any physiological changes that cutaneous, muscular, or vestibular input could impart during the same passive movements involved in preparing the participant for the spinal manipulative procedure. It also acted as a control for the time taken to carry out the spinal manipulation intervention and the stimulus necessary to collect the dependent measures of the study. Following the experiment, subjects were told that there was an experimental and sham intervention and they were asked which intervention group they believed they were in. Most participants in the control group indicated that they believed they had received a chiropractic intervention and many of them also indicated that they felt better after the sham intervention. 

### 2.2. Data Analysis

**Figure 2:**
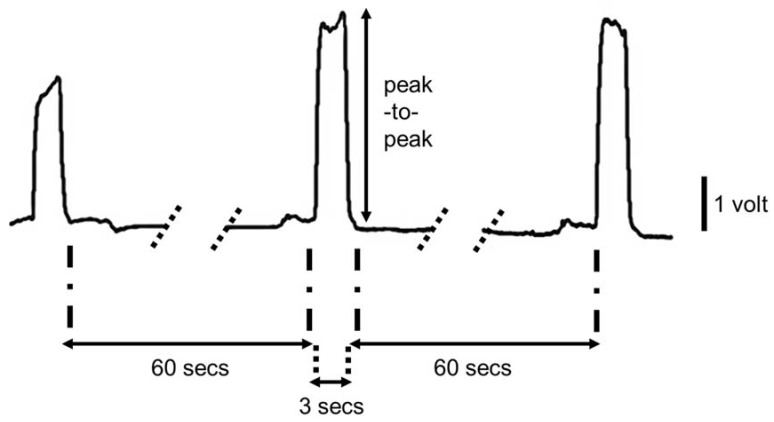
A sample recording is shown from a subject is shown. Each peak belongs to a maximal bite trial that lasts about 3 s, with about 60 s interval between each attempt.

The bite force recordings were amplified 50 times and sampled at 2000 Hz (CED Limited, Cambridge, UK). For the analysis, peak-to-peak values of each bite force record were measured using Spike2 7.03 (Cambridge Electronic Design, Cambridge, UK) software. A drawing of a sample recording is shown in Figure 2. The maximum peak-to-peak value from the three bite attempts was converted to “Newtons” and the percentage change was calculated for the pre- to post and post 1-week assessments.

### 2.3. Statistical Analysis

Repeated measures ANOVA’s with factors of INTERVENTION (spinal manipulation and Sham) and TIME (Pre, Post, 1-week) were used to assess for intervention effects immediately post-intervention and at 1-week follow up. Two ANOVAs were used due to a large number of dropouts between the post-intervention and 1-week follow-up assessment sessions. Post hoc *t*-tests were used to assess for within-group changes where interaction effects were observed. Significance was set at *p* < 0.05 for all statistical tests.

## 3. Results

Immediately post-intervention there was a significant INTERVENTION × TIME interaction for the maximum bite force measurements (F (1,26) = 5.96, *p* = 0.02). Post hoc analysis revealed a significant increase in bite force in the chiropractic group of 11.0% (±18.6%) post-intervention (*p* = 0.04). Immediately after the sham intervention bite force in the control group decreased by 2.3% (±9.0%, *p* = 0.20).

**Figure 3:**
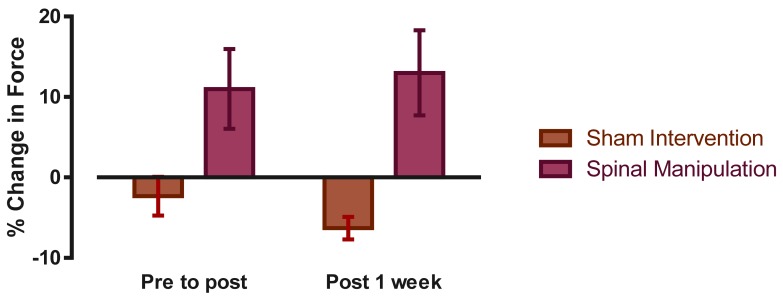
Average percentage change in total bite force values (mean ± SE) for both spinal manipulation and Sham (control) experiments.

At the 1 week follow up assessment the interactive effect remained (F (1,10) = 10.92, *p* < 0.01) with bite force in the chiropractic group remaining 13.0% (±12.9%, *p* = 0.04) stronger than baseline. In the control group bite force decreased by 6.3% (±3.4%, *p* = 0.01) at the 1-week follow-up. See Figure 3.

## 4. Discussion

The major finding of this study was that chiropractic spinal manipulation increased maximum bite force immediately after the intervention and the increase in bite force remained at 1-week follow-up. This is the first study to show that a single session of chiropractic spinal manipulation can increase jaw bite strength compared to a sham intervention.

This immediate increase in jaw bit force of 11% post spinal manipulation was unlikely to be due to the placebo effect, as all subjects were naïve to chiropractic, and most of the subjects did not know which intervention was real upon questioning after both interventions. The 2.3% decrease in maximum bite force after the sham intervention may have been due to fatigue from maximum biting on the mold, or simply due to random variations in maximum efforts.

From the existing literature, it is known that age [10,12], gender [10], the condition of temporomandibular joint [11,27], as well as unilateral or bilateral clenching [28] are known factors to influence maximal bite force. The current study now also suggests that cervical spine function can influence maximal bite force. The effort with which the subject’s bite would also influence maximum bite force, and for this reason the study was conducted in Turkey, where chiropractic is relatively unknown, to enable a more effective sham intervention. As no increase in strength occurred following the sham intervention, the effort is unlikely to have been the reason the subjects’ bite force increased after the spinal manipulation.

Only a small number of studies have previously investigated changes in muscle strength following spinal manipulation [4,29,30,31,32]. Increases in lower limb muscle strength in subjects with subclinical pain following chiropractic spinal manipulation has been reported [4]. An increase in lower limb strength in elite athletes that lasted 30 min post spinal manipulation was shown [6]. Chilibeck, et al. [33] reported that in subjects with imbalances in lower limb muscle strength, spinal manipulation resulted in increased muscle strength of hip abductors in their weak leg [33]. Botelho and Andrade [31] reported increases in grip strength in a group of national level judo athletes following spinal manipulation [31]. However, no changes in handgrip strength in asymptomatic basketball players following spinal manipulation was found [32]. Changes in strength following spinal manipulation may, therefore, vary both depending on the muscle tested as well as population characteristics.

In two of these previous studies that showed lower limb muscle maximum voluntary strength increases after chiropractic spinal manipulation [4,6] H-reflex excitability and V-waves were also recorded. Both studies showed increases in maximum plantarflexion force and significant increases in the cortical drive to the plantar flexors (i.e., V-wave) following spinal manipulation, and that both these measures significantly decreased after the control intervention [4,6]. The decrease in MVC force and cortical drive (V-wave amplitude) after the control intervention is most likely due to fatigue occurring due to the repeated maximum contractions the subject needed to perform to enable V-wave recordings, as highlighted by the authors of these studies. The increase therefore seen following the spinal manipulations was, therefore, most likely because of the increased cortical drive to the muscle. The authors argued this because only minor or no changes in the H-reflex were observed in these studies [4,6]. Other research also suggests that such changes in strength may be caused by supraspinal changes [34,35].

It has previously been proposed in the literature that chiropractic spinal manipulation has a central neural effect. This is because multiple studies have shown that spinal manipulation of dysfunctional spinal segments can impact somatosensory processing, sensorimotor integration, and motor control as mentioned in the introduction [1,2,3,36,37,38,39]. This current study supports this notion, as spinal manipulation appears to alter maximum biting force in this group of subjects. This study, therefore, supports the growing body of research that suggests chiropractic spinal manipulation’s main effect is neuroplastic in nature that affects cortical excitability [1,2,3,5].

Spinal dysfunction, even mild, recurrent spinal dysfunction, has been shown to be associated with maladaptive neural plastic changes, such as alterations in elbow joint position sense [39], mental rotation ability [40], and even multisensory integration [41], suggesting spinal dysfunction can alter the brains inner body schema and maps of the body and the world around us. This may be because spinal manipulation has been shown to change both cerebellum-M1 processing [42] as well as prefrontal cortex processing [5]. In the current study, the subjects’ mild spinal dysfunction may have altered the somatosensory input from the neck to the brain centers involved in sensorimotor integration and motor control of the jaw, and that adjusting these dysfunctional segments therefore impacted on these same central regions altering the maximum bite force the subjects could perform.

Knowing that spinal function can have an impact on jaw function has functional implications for patient populations. It is possible that chiropractic spinal manipulation may influence the clinical outcomes for patients with TMJ disorders, as has been suggested by individual case studies [7]. Future research should further investigate this using properly powered clinical trial designs.

This current study was a small basic science study, thus there are several limitations to be aware of. Future research could ensure group randomization as well as ensuring equal distribution of age and gender in each group, and longer data collection windows so adequate follow-up can be undertaken. Larger groups would also be beneficial as would including clinical populations that have TMJ disorders.

## 5. Conclusions

This study found that chiropractic spinal manipulation significantly increased subjects’ maximal bite force and this increase was still evident 1-week post spinal manipulation

In this study, the sham intervention had no significant effect on the maximal biting force. This study supports the concept that spinal manipulation has a central neuroplastic effect. The clinical implications of this study should be followed up in populations that have TMJ disorders.
